# The antibacterial efficacy of a foam mouthwash and its ability to remove biofilms

**DOI:** 10.1038/s41405-018-0005-5

**Published:** 2018-09-27

**Authors:** Siân B. Jones, Nicola X. West, Pavel P. Nesmiyanov, Sergey E. Krylov, Vera V. Klechkovskaya, Natalya A. Arkharova, Svetlana A. Zakirova

**Affiliations:** 1 Clinical Trials Unit, Bristol Dental School, Bristol, UK; 2 Fundamental Medicine and Biology Department, Volgograd State Medical University, Volgograd, Russian Federation; 3 Department of Immunology, Biological Faculty, Lomonosov Moscow State University, Moscow, Russian Federation; 4 LLC BITECA, Odintsovo, Moscow region, Moscow, Russian Federation; 5 Shubnikov Institute of Crystallography FSRC “Crystallography and Photonics” RAS, Moscow, Russian Federation; 6 SPLAT Cosmetics LLC, Moscow, Russian Federation

## Abstract

**Objectives/Aims:**

To evaluate the antimicrobial activity of a newly developed foam mouthwash containing a modified lactoperoxidase system in vitro.

**Materials and methods:**

Biofilms of five bacterial species were developed on hydrophobic and hydrophilic surfaces whilst salivary-based biofilm was grown on tooth enamel. Each surface was exposed to the foam mouthwash or saline in vitro. Optical density and scanning electron microscopy (SEM) was used to determine retention of the biofilm following 5 or 30 s exposure time.

**Results:**

The foam mouthwash was active against biofilms formed by *S. aureus*, *K. rhizophila*, *M. thailandicus, E. coli*, and *C. violaceum* and eliminated significant amount of biofilm from each surface; immature 4 h biofilm was less resistant than 24 h biofilm. A 30 s rinse showed best performance, with removal of up to 66% of biofilm from the hydrophilic surface. SEM imaging confirmed oral biofilm removal from the enamel surface after a 5 s rinse with the foam mouthwash.

**Discussion:**

Foam mouthwash demonstrated a significant impact on growing biofilm when compared against saline solution. Growing biofilms were more susceptible to the action of the foam mouthwash, which justifies after-meal use of the mouthwash when traditional dentifrices may not be accessible.

**Conclusions:**

Foam mouthwash can be a convenient on-the-go format of oral care products that can be used after meals or when needed to reduce the risk of biofilm-associated oral conditions.

## Introduction

Biofilms are complex microbial communities characterized by cells attached to the substrate surface, to interfaces or to each other and are embedded in an extracellular polymeric matrix which they have produced.^1–3^ Mixed culture microbial biofilms comprise dental plaque that can be beneficial to the host, but biofilms are often associated with diseases.^4^ Tightly adherent mature biofilms are a primary cause of caries and their formation needs to be prevented.^5^ Clinically biofilms form on native tissues such as oral mucosa and teeth and often cause chronic infection of dental implants.^6–8^ The behavior of microbes in a biofilm can differ significantly from the behavior of the same organism studied in planktonic conditions.^3^ Most studies on dentifrices focus on bacteria commonly isolated from dental plaque, however these studies do not take into consideration the different adhesion mechanisms of some bacterial species and the beneficial role of some microorganisms implicated in biofilm formation.^9,10^

Latest data on human microbiome suggest that best oral care practices should not seek complete elimination of biofilms but to control their formation without damaging the protective pellicle.^11^ One of the possibilities to beneficially alter the ecology of dental biofilm is the use of lactoperoxidase system.^12^ Lactoperoxidase systems have been incorporated into dental healthcare products such as dentifrice and mouthwashes since the 1980s.^13^ The byproduct of lactoperoxidase is hypothiocyanate which acts as a natural antibacterial agent.^12^ The management of biofilm related conditions can be problematic as the structure and composition of the biofilm itself offers protection against antimicrobial agents and frequently conjunctive mechanical biofilm disruption is required to enable surface disinfection.^14^

The effectiveness of over-the-counter toothpastes is without question but toothpastes have limited portability and are not well suited for daytime use. At the same time, maturing biofilms are most susceptible to external impacts, which justifies the use of oral care products during the day, especially after meals. Considerable research effort is currently being spent developing portable products for on-the-go use. Conventional mouthwashes cannot be attributed to easy-to-use formats because of relatively large package volumes and high consumption volume (about 20 mL). A novel format of mouthwashes has recently been developed in the form of a foam mouthwash, which is portable and convenient to use. The aim of the present in vitro study was to investigate the antibacterial effect of foam mouthwash against biofilms formed on the surfaces mimicking real-life conditions. The null hypothesis being that a foam mouthwash has no greater effect than saline at removing biofilm.

## Materials and methods

This study was conducted in two parts. One in which artificial surfaces were used for biofilm quantification and another where extracted teeth were used for visualization by scanning electron microscopy (SEM).

### Solutions and products used

“Splat Oral Care Foam Raspberry 2 in 1™” (SPLAT Cosmetics, Russia) mouthwash was used. This product contains modified lactoperoxidase system [potassium thiocyanate, lactoferrin, lactoperoxidase, glucose oxidase, glucose pentaacetate, *Glycirrhizia Glabra* root extract] named as the registered trademark Luctatol™ and detergents, designed to provide cleaning and antibacterial action. Sterile saline was used as control.

### Biofilm retention study

Several strains of bacteria to model biofilm formation on the surfaces were used. Gram-positive *Staphylococcus aureus* 209P, *Kocuria rhizophila* 4А-2ZH (also known as *Micrococcus luteus*), *Micrococcus thailandicus* НВ, Gram-negative *Escherichia coli* К-12 and *Chromobacterium violaceum* WT were obtained from the strain collections of the research center of biotechnology RAS (Moscow, Russia). These bacteria were selected on the basis of their different adhesion mechanisms and different extracellular matrix structure. Bacteria were seeded onto solid LB medium (Carl Roth GmbH, Karlsruhe, Germany) with consequent transfer of single colonies into liquid LB medium and incubated for 24 h at 30 °C. Teflon cubes (*n* = 10 for each condition) were used for biofilm formation, as described in Plakunov et al.^15^ Cubes were immersed in LB medium containing 50 µL of 24 h culture diluted to 2 × 10^9^ CFU/mL and incubated for 4 and 24 h at 30 °C on a MaxQ 4000 rocker platform (150 rpm) (Thermo Scientific, Waltham, MA, USA) allowing for biofilms to be deposited both on the cubes and vial glass internal surface (to mimic tooth enamel hydrophobic and hydrophilic properties). LB without bacteria was used as a blank. After incubation, cubes and vials were washed with sterile saline and washed again with 3 mL mouthwash or saline for 5 or 30 seconds using a vortex (Micro–Spin FV-2400, BioSan, Lithuania) set at 3000 rpm. Unwashed samples were used as a control and the amount of biofilm retention on the treated surfaces was quantified as a % difference from this value. Biofilms were then stained with Crystal Violet (Sigma, St Louis, MO, USA) for 30 min. After washing with distilled water the biofilms were extracted from cubes and vials surfaces with 96% EtOH for 1 h. Optical density of the resulting solution was measured at 590 nm using a Statfax 2100 optical density reader (Awareness Technology, Palm City, FL, USA).

### Biofilm retention and SEM imaging study

The ethics committee of Shubnikov Institute of Crystallography FSRC “Crystallography and Photonics” RAS approved the experimental protocol for the use of human teeth and saliva (Protocol #7). Extracted mandibular central incisors (*n* = 10) were cleaned with a hard toothbrush and high-abrasion silica (Sorbosil ac33®, Surfachem, Leeds, UK) before being ultrasonically cleaned in distilled water for 4 h at 35 kHz (Sonoswiss SW3H, Sonoswiss AG, Ramsen, Switzerland). Teeth were air-dried at room temperature. After this cleaning method the teeth showed no signs of bacterial contamination under SEM.

After primary SEM imaging the teeth were transferred into 20 mL A. C. Broth (Sigma, St Louis, MO, USA) containing pooled human saliva and incubated for 48 h at 37 °C on a MaxQ 4000 rocker platform. After 24 h, а change in pH from 6.8 to 3.5 was noted and the medium was replaced with fresh medium and incubated for another 24 h, as described by Zhou et al.^16^

Saliva was pooled for wild-type organisms from five subjects who refrained from all oral hygiene procedures overnight. Pooled tongue scrapings from five different individuals were also obtained using tongue cleaners which were rinsed with 0.01% peptone. The samples were then centrifuged for 10 min at 10,000 × *g* (MicroCL 21R, Thermo Scientific, Waltham, MA, USA), following which the pellets were resuspended in A. C. Broth to a final volume of 20 mL. Pre-study criteria for saliva pooling was that donors did not consume any antibiotic products in the month previous to the sampling day, refrained from using an antiseptic mouthwash in the week before the study, avoided any oral hygiene measures in the morning when the sample was taken and abstained from food or drink intake for at least 2 h prior to donating saliva. Second SEM analysis was made after the incubation to detect biofilm formation.

Five incisors were agitated using a vortex (Micro–Spin FV-2400, BioSan, Lithuania) set at 3000 rpm in 5 mL foam mouthwash for 5 s and 5 incisors were incubated in sterile saline for 5 s. After incubation the teeth were rinsed with distilled water and air-dried at room temperature before third SEM analysis.

SEM was carried out on the surfaces of uncoated samples attached to carbon tabs on a Scios field emission scanning electron microscope (FEI, Eindhoven, The Netherlands), operated at an accelerating voltage of 1.0 kV. Samples were placed into the SEM sample holder at the same orientation each time so that the same area could be identified and standardized. Imaging was performed at a working distance of 5–7 mm. Images taken at ×8000 and ×30,000 magnification was used for analysis.

### Statistical analysis

The statistical analysis was performed using IBM SPSS 22 for Windows. To detect statistically significant inhibition in an unbiased approach, we performed the non-parametric sign-test. The level of significance was set at 0.05.

## Results

### Biofilm removal

Results on the *E. coli* and *M. thailandicus* biofilm removal are presented in Table 1. Our data suggest that foam mouthwash was superior to saline in the removal of both growing and mature biofilms. However, in most cases 5-s rinse was not enough to remove mature biofilm. Similar results were obtained with *S. aureus* and *K. rhizophila* biofilms. However, in all cases foam mouthwash reduced growing biofilm significantly after a 30-s rinsing procedure, being most effective on glass surface. *C. violaceum* biofilms was most resistant to rinsing while *E. coli* was most susceptible with up to 2/3 growing biofilms removed (34.4% retention) after a 30-s rinse.

**Table 1:** Growing (4 h) and mature (24 h) biofilm retention (O.D. 590, 5 s/30 s, data presented as median, % to control (Control = 100%))

	Mouthwash (*n* = 50)				Saline (*n* = 50)			
	4 h		24 h		4 h		24 h	
	Glass	Teflon	Glass	Teflon	Glass	Teflon	Glass	Teflon
*E. coli* K-12	51.2*/34.4*	63.0*/48.3*	90.7/66.3*	98.3/73.0*	90.0/90.1	92.3/88.0	96.0/95.4	98.5/96.0
*Control O.D*. _*590*_	0.64	0.79	0.77	0.58	0.79	0.87	0.81	0.52
*M. thailandicus*	71.0*/44.2*	83.0*/53.8*	98.8/76.5*	98.5/77.0*	93.6/92.1	98.3/98.0	101.0/97.7	98.8/97.0
*Control O.D*. _*590*_	0.75	0.84	0.73	0.74	0.58	0.64	0.57	0.59
*S. aureus* 209P	61.5*/54.2*	72.8*/56.8*	68.9*/59.2*	75.3*/68.1*	91.5/80.7	84.4/72.7	95.9/89.4	94.8/77.7
*Control O.D*. _*590*_	0.81	0.79	0.57	0.66	0.54	0.79	0.72	0.63
*K. rhizophila*	59.3*/46.0*	89.9/82.6*	67.4*/52.6*	93.1/84.7*	98.9/94.4	99.9/95.6	98.3/95.5	101.2/98.2
*Control O.D*. _*590*_	0.54	0.74	0.72	0.76	0.71	0.75	0.54	0.86
*C. violaceum*	80.1/71.2*	86.1/83.3*	85.7/76.1*	91.1/86.9	98.2/94.0	99.6/98.3	100.5/95.7	97.7/97.0
*Control O.D*. _*590*_	0.63	0.87	0.86	0.54	0.85	0.64	0.59	0.52

Correspondent median control O.D. values are presented below each dataset

*p < 0.05 compared to saline

### Scanning electron microscopy

SEM images of the enamel surface captured prior to biofilm formation (not shown) appeared smooth and clean. There were no bacteria, pellicle, particles, or deposits. Surfaces without carious lesions or demineralization were used.

**Fig. 1:**
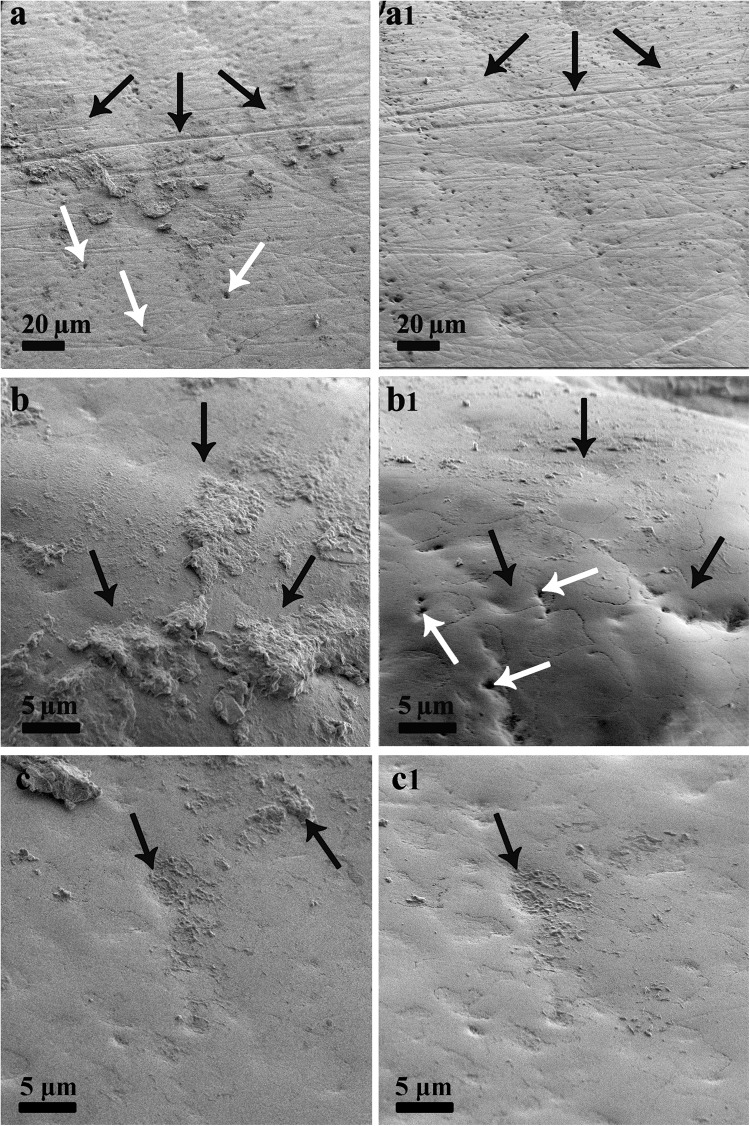
Representative SEM images of enamel surfaces. **a**–**c** After biofilm formation, before foam or saline rinsing; a1, b1—after foam rinsing, c1—after saline rinsing. Black arrows indicate biofilm location site. White arrows indicate erosive pits

After incubation with pooled saliva, biofilms were formed at the enamel surfaces and enamel erosions were present. As shown in Fig. 1, a 5-s rinse with foam mouthwash led to significant removal of biofilms compared to saline control.

## Discussion

The present study investigated the effectiveness of foam mouthwash rinsing on biofilm retention on different surfaces. The study design was developed to explore the capability of foam mouthwash to remove biofilms of bacteria with different adhesion properties from diverse surfaces—hydrophilic (glass), hydrophobic (Teflon cubes), and tooth enamel. Foam mouthwashes are a relatively new type of oral care product on the market and there are no previously published studies on the efficacy of such products. Fluoridated foams used for caries control have previously been shown to reduce the case of caries development when compared to the more commonly used fluoride gels.^17^ Foams are deemed advantageous because of the reduced amount of volume that is required to achieve the same outcome as when fluoridated gels are used.^18^ Most mouthwashes claim the ability to clean teeth after 5–30 s swishing. This ability is mostly mediated by surfactants that change the permeability of bacterial cell membrane with subsequent cell lysis.^19,20^ The presence of the foam pump enables output of the mouthwash liquid in the form of a foam with bubble size ranging from 0.05 to 0.5 mm. This allows a reduced mouthwash volume to be used whilst more importantly, employs the physical phenomenon of bacterial cell damage by the bubble rupture at the gas–liquid interface.^21,22^

In both parts of this study the foam mouthwash demonstrated a significant impact on growing biofilm when compared against saline solution. The first part of the study showed that *E. coli* was most susceptible and *C. violaceum* was most resistant to the action of mouthwash. As expected, growing, or immature 4 h biofilms were more susceptible to the action of the foam mouthwash, which justifies after-meal use of the mouthwash when traditional dentifrices may not be accessible. In addition, since recommended use of mouthwash does not imply water rinsing, it is expected that the lactoperoxidase system within the foam mouthwash will continue to work for a certain period of time, thus preventing colonization and cariogenic action of acid-producing bacteria.^12,23^

The results obtained from the second part of the study where enamel was used as substrate again showed that the foam mouthwash performed better than saline at removing biofilm. The biofilm formed on the enamel surface was a mature biofilm as the method of growing the biofilm meant that a 48 h biofilm was developed. During the development of the biofilm, erosive pits also formed on the surface. The erosive pits were not altered by the exposure to the foam mouthwash. The pH of the foam mouthwash was 7.4 which is similar to Bioténe Oral Balance which also contains lactoperoxidase and has previously been shown to not cause enamel erosion.^24^ The SEM images show that the biofilm was not uniformly developed over the surface but that the amount removed following a 5 s rinse with the foam mouthwash was greater than with saline. Increasing the exposure time to 30 s would most likely remove more of the formed biofilm as evidenced in the first part of the study. Interestingly, the amount of mature (24 h) biofilm removed from the glass and Teflon substrates after a 5 s treatment with the foam mouthwash was not great. Only the *S. aureus* 209P and the *K. rhizophila* showed significant removal compared to saline. This highlights the importance of recognizing that differently formed biofilms can provide varying results and that the type of substrate used can also determine the amount of biofilm retention.

## Conclusions

In conclusion, the results from this study can reject the null hypothesis as the foam mouthwash was significantly more effective than saline at removing biofilm from each of the surfaces investigated. The foam mouthwash can be recommended as a convenient, additional oral hygiene device for daily after meal use.
